# A scoping review of the Trauma Recovery Center model for underserved victims of violent crime

**DOI:** 10.3934/publichealth.2024064

**Published:** 2024-12-16

**Authors:** Annette M. Dekker, Jennifer Wang, Jason Burton, Breena R. Taira

**Affiliations:** 1 Department of Emergency Medicine, David Geffen School of Medicine, University of California, Los Angeles, California, USA; 2 Department of Emergency Medicine, Olive View-UCLA Medical Center, Sylmar, California, USA; 3 Department of Emergency Medicine, University of Michigan Medical School, Ann Arbor, Michigan, USA; 4 UCLA Library, University of California, Los Angeles, California, USA

**Keywords:** Trauma Recovery Center, crime victim, health disparities, psychosocial support, social services, outcome assessment

## Abstract

Victimization in the United States is common and has long lasting negative impacts for individuals, often disproportionately impacting those of color and from low socioeconomic communities. The Trauma Recovery Center (TRC) model aims to provide comprehensive mental health and wrap-around case management services for underserved victims of crime. Following PRISMA-ScR guidelines, we sought to further our knowledge about the impact of the TRC model. Twelve studies met the inclusion criteria. Studies were based at three sites. Access to treatment ranged from 55.7% to 72.3%; treatment completion rates ranged from 40.4% to 43.0%. Individuals who completed mental health services showed improvement in PTSD, anxiety, and depression symptoms, while experiencing lower rates of injury recidivism. Several studies demonstrated improvement in mental health symptoms and social needs in individuals from underserved communities. Researchers should focus on expanding and diversifying upon current knowledge to better understand the impact of the TRC model.

## 1. Introduction

Victimization in the United States is common and has long lasting negative impacts for both individuals and communities. Researchers estimate that more than 3.5 million individuals per year are victims of violent crime in the United States [1]. These victimizations can have substantial impacts on survivors' physical and mental health [2], which in turn affects relationships with family and friends, performance at work and school, likelihood of substance use, and risk of future victimization [3],[4]. This violence disproportionately impacts underserved individuals, particularly those of color and from low socioeconomic communities. The risk of experiencing serious violence is 1.2 to 1.5 times greater for Latinx individuals and 1.5 to 2 times greater for black individuals compared to their white counterparts [5]. Despite higher rates of victimization, these individuals are less likely to receive mental health and social services following a crime, often as a result of structural inequities and decreased access to essential resources [6].

The Trauma Recovery Center model, first conceptualized and implemented at the University of California, San Francisco (UCSF) in 2001, is a public health intervention designed to address the gaps in services for victims of crime from underserved populations by providing comprehensive mental health and wrap-around case management services to all [7]. The model is not intended to replace traditional mental health services, but rather provide intensive and comprehensive trauma-informed wraparound services for victims of violent crime that may otherwise not have access to care. The model can be hospital- or community-based and can receive referrals from broad range of sources, including but not limited to hospitals, schools, law enforcement, and local community organizations.

All TRCs include 11 core elements as dictated by the TRC handbook including, but not limited to: Serving survivors of all types of violent crimes, regardless of immigration status, and inclusive of those with complex challenges; assertive outreach; comprehensive mental health; clinical case management; multidisciplinary team; coordinated care tailored to individual needs; use of trauma-informed and evidence-based practices; goal-driven; and accountable services [8]. Assertive outreach – defined as outreach through text messages, phone calls, letters, home visits, or community visits to those lost to contact or not well-engaged – is utilized to engage survivors of violent crime and communities that may experience barriers to traditional services. In juxtaposition to other models of care, the provision of case management alongside mental health services in the TRC model ensures that basic needs – such as safety, housing, and food security – are addressed to remove barriers to engaging with recovery. The TRC model necessitates the use of evidence-based practices, defined in the TRC handbook as “those that have been identified by nationally or internationally recognized trauma experts (such as the American Psychological Association, the U.S. Department of Defense, SAMHSA, and the International Society for Traumatic Stress Studies) as having demonstrated clear research outcomes to support their use for the treatment of trauma” [8]. Examples of evidence-based practices recognized by the TRC handbook include motivational interviewing, seeking safety, cognitive behavioral therapy, narrative exposure therapy, prolonged exposure therapy, and cognitive processing therapy [8]. Separate from evidence-based psychotherapy, the model also encourages a culture of trauma-informed care, defined by the Substance Abuse and Mental Health Services Administration (SAMHSA) as “a program [that]... realizes the widespread impact of trauma and understands potential paths for recovery; recognizes the signs and symptoms of trauma in clients, families, staff, and others involved with the system; and responds by fully integrating knowledge about trauma into policies, procedures, and practices, and seeks to actively resist re-traumatization” [9]. Finally, the TRC model assigns a primary clinician, also known as a single point of contact, to each survivor to reduce the burden of survivors having to engage with multiple providers at a time when they may have limited capacity [8].

Prior to 2017, the TRC model had been implemented only at five sites in California [10]. Over the past six years, the TRC model has expanded exponentially. At the time of this writing, the TRC model has been implemented at 53 centers across 12 states, with 14 of these sites opening since 2022 [10]. In many states, funding for TRCs is coded into legislation. In California alone, $22 million was allocated to fifteen TRCs to provide services from 2023 to 2025 [11].

Despite the widespread adoption of the TRC model, little is known about the implementation or impact of the model outside of the original TRC at UCSF. Limitations in research may be due to the recent expansion of the model, as well as limited funding dedicated to site-led evaluations. To the authors knowledge, no prior scoping reviews have been conducted that examine research for studies that evaluate the TRC model. The goal of this scoping review is to describe the existing evidence for the TRC model.

## 2. Materials and methods

Due to the anticipated low number of studies and evolving landscape of Trauma Recovery Centers, we chose a scoping approach for this review. We followed the Preferred Reporting Items for Systematic reviews and Meta-Analyses extension for Scoping Reviews (PRISMA-ScR) guidelines [12].

### 2.1. Eligibility criteria for study consideration

Studies that entailed evaluating services provided at a Trauma Recovery Center were sought for this review. The following criteria were used to assess study inclusion: (1) Original research published between January 1st, 2001 and March 31st, 2022; (2) research based in a Trauma Recovery Center (as defined by the UCSF model initiated in 2001); and (3) an evaluation of TRC services including but not limited to implementation and outcome metrics.

### 2.2. Information sources and search strategy

Search terms related to victims of crime and psychosocial interventions were developed with the assistance of a librarian (author JB) (Table 1). To identify potentially relevant literature, the PubMed, Embase, and PsycInfo databases were searched on June 22nd, 2022. An additional simplified search with the search terms “Trauma Recovery Center” was performed in Google Scholar to capture grey literature not previously identified. Citations of search results were reviewed for additional studies. Finally, experts were asked to identify additional key articles that were not captured in the primary search.

**Table 1.** Search terms for scoping review.

**Population AND**	**Trauma Recovery Center AND**	**Intervention**
(“survivors” OR survivor* OR victim*) AND (“violence” OR “interpersonal violence” OR “trauma” OR “crime”)	(“trauma recovery” OR “psychosocial services” OR “case management” OR “health service”)	(“psychosocial support” OR “psychosocial needs” OR “psychotherapy” OR “psychological services” OR “social service” OR “social work” OR “compensation fund” OR “treatment”)

### 2.3. Screening and eligibility

**Figure 1.**
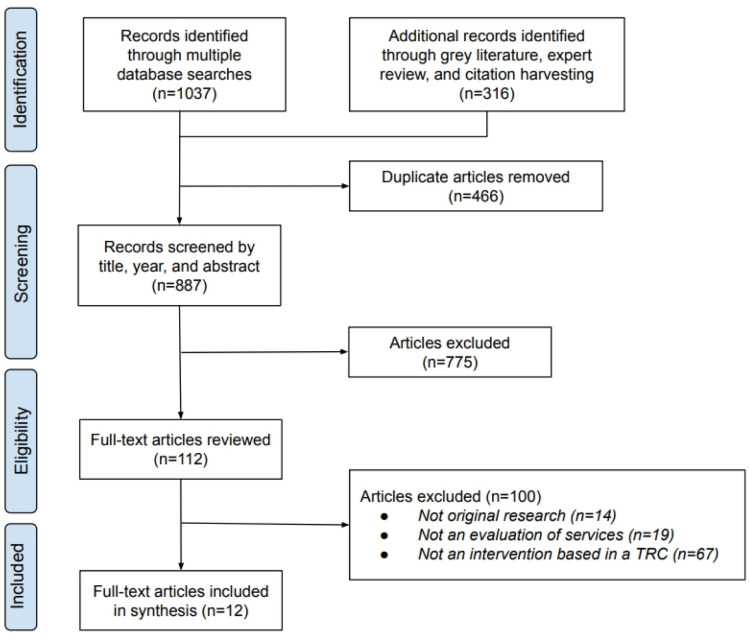
PRISMA-ScR flow diagram.

One author (JB) extracted the title, year of publication, and abstract of all identified articles. Duplicates were removed. Identified articles underwent a two-step review process (Figure 1). First, two authors (AD and JW) independently screened the title, year of publication, and abstract of all identified studies to determine whether the study met the eligibility criteria outlined above. Studies identified by either author were advanced to full-text review. Second, two authors (AD and JW) independently reviewed the full text of each study to determine whether the study met eligibility criteria. A third author (BT) was available for resolution of discordance for final study inclusion.

### 2.4. Collecting, summarizing, and reporting of results

Pre-identified elements were extracted and entered into a data extraction table by one author (AD) and verified by a second author (JW). Extracted data include authors, study location, population, methodology, outcome measures, results, and whether impact on specific marginalized populations were addressed, and if so, which populations.

## 3. Results

### 3.1. Study location and population

Twelve articles met the criteria (Table 2) [13]–[24]. Two of the studies were conducted at the University of California, San Francisco Trauma Recovery Center (UCSF TRC) in San Francisco, California, formerly known as San Francisco General Trauma Recovery Center [13]–[14]. Eight of the studies were based at Long Beach Trauma Recovery Center (LBTRC) in Long Beach, California [15]–[22]. Finally, two of the studies were conducted at the Victims of Crime Advocacy and Recovery Program (VOCARP), known as the MetroHealth Trauma Recovery Center, located at MetroHealth Medical Center in Cleveland, Ohio [23],[24]. Two studies were randomized control trials [13],[15]; the remaining studies were retrospective cohort studies [14],[16]–[24].

Individuals included from the UCSF TRC studies received services from 2001 to 2006 [13],[14]. The studies were restricted to individuals aged 18 years and older who received emergency medical treatment at San Francisco General Hospital. The population in these two studies was predominantly male (75.1% and 71.7%) and black (51.7% and 49.5%) with a mean age of approximately 37 years [13],[14]. Of note, victims of sexual assault were excluded due to an alternative county program at the time of the studies.

Participants included from the LBTRC studies received services from April 2014 to March 2020 [15]–[22]. One study was restricted to individuals less than 18 years [17]; the other studies included adults ages 18 and older [15],[16],[18]–[22]. The study population across the eight studies were predominantly female (ranging from 60.2% to 100.0%) and Latinx (ranging from 43.7% to 61.7%). For studies restricted to adults, the mean age ranged from 34.4 to 35.9 years [17]; for the study restricted to youth, the mean age was 11.5 years [15],[16],[18]–[22].

Individuals included from the VOCARP studies in Ohio received services from March 2017 to December 2018 [23],[24]. Study participants included any individual who presented to the emergency department for traumatic injury; there was no exclusion criteria. Individuals who received VOCARP services were predominately male (55.6%) and black (54.3%). The mean age for individuals who received VOCARP services was 34.4 years.

All of the studies used a program evaluation framework. Upon reviewing the articles, the reported metrics were categorized as one of the following: 1) Process metrics of client services; 2) outcome metrics for social and mental health needs; or 3) impact of TRC services on inequities in process and outcome metrics.

### 3.2. Process metrics: Treatment access, initiation, and completion

Six of the studies included treatment access, initiation, or completion as the primary study outcomes (Table 2) [14],[17],[18],[20]–[22]. In three studies, it was found that the percentage of individuals who accessed treatment, defined as completing screening or intake interview, compared to all individuals who were referred to care was 55.7% [20], 68.4% [21], and 72.3% [14]. Treatment initiation rates, defined as engaging in at least one psychotherapy or case management session, were 44.0% [20], 60.0% [14], 64.2% [22], 69.5% [17], and 72.0% [21]. Two studies assessed treatment completion defined as either completing at least eight sessions [17] or nine sessions [21]; completion rates were 43.0% and 40.4%, respectively. Another study showed that individuals who received person-centered therapy (PCT) had the lowest proportion of dropout (<9 sessions, 41.75%) compared to cognitive behavior therapy (CBT) (56.82%) or eclectic therapy (61.05%) (p < 0.05) [18].

### 3.3. Outcome metrics: Victim compensation, injury recidivism, and change in mental health symptoms

Seven studies addressed outcome metrics related to receiving TRC services (Table 2) [13],[15],[18],[19],[21],[23],[24]. In one study, a randomized controlled trial was used to evaluate the rate of victim compensation claim submissions [13]. Of those who were randomized to TRC services, 55.9% (n = 189) filed victim compensation claims in comparison to 23.0% (n = 47) of individuals receiving usual care (p ≤ 0.001). Of those who filed victim compensation claims, 78.3% (n = 148) of those receiving TRC services successfully received compensation, compared to 91.5% (n = 43) of those receiving usual care (p = 0.04).

Two studies evaluated the rate of recidivism in individuals who received TRC services in comparison to those who did not receive TRC services [23],[24]. Recidivism was defined as presenting to the emergency department or clinic for a new violence-related injury. When comparing those who received TRC services compared to those who did receive TRC services, no difference in injury recidivism was found (10.9% vs. 8.5%, p = 0.33) [23],[24]. However, for individuals enrolled in TRC services, those who used mental health services had lower rates of recidivism (4.4%) compared to those who did not enroll in mental health services (11.7%, p = 0.016) [24].

Four studies evaluated changes in symptoms of posttraumatic stress disorder (PTSD), depression, and anxiety as measured by clinical tools, including the PTSD Checklist for DSM-5 (PCL-5), Brief Symptom Inventory-18 (BSI-18), SDS (Sheehan Disability Scale), Life Events Checklist (LEC-5) [15],[18],[19],[21]. In these studies, changes in symptoms across sessions, as well as changes in symptoms by clinical demographics and type of treatment received, were evaluated.

In one study, a decrease in symptoms of PTSD, depression, and anxiety from session one to session to nine was demonstrated [21]. In addition, the percentage of individuals who met clinical cutoff for PTSD (defined as ≥33) improved from 72.6% (n = 249) at session one to 32.2% (n = 111) at session nine (p < 0.001). For individuals who completed depression symptom assessments, 68.6% (n = 229) met criteria for depression (defined as T score ≥ 68) at session one, compared to 41.6% (n = 139) at session nine (p < 0.001). Finally, 68.3% (n = 228) of individuals screened positive for anxiety (defined as T score ≥ 68) at session one, compared to 46.1% (n = 154) at session nine (p < 0.001).

In one study, the association of race/ethnicity with changes in symptoms of PTSD, anxiety, and depression from week one to week six was evaluated [19]. It was found that at six weeks, white participants had an increased likelihood of PTSD compared to Latinx participants (OR = 0.32, 95% CI, 0.11, 1.00, p < 0.05), increased likelihood of depression compared to individuals who identified race as other (OR = 0.17, 95% CI, 0.04, 0.78, p < 0.05)), and increased likelihood of anxiety compared to black participants (OR = 0.07, 95% CI, 0.13, 0.38, p < 0.01), Latinx participants (OR = 0.09, 95% CI, 0.02, 0.42, p < 0.01), and individuals who identified race as other (OR = 0.05, 95% CI, 0.01, 0.32, p < 0.01).

Differences in changes in symptoms by treatment type were evaluated in three studies [15],[18],[19]. In one study, there was no statistically significant difference in PTSD, depression, anxiety, or somatization symptoms between prolonged exposure therapy (PE) compared to person-centered therapy (PCT) (p < 0.05) [15]. In another study, there was no difference in PTSD symptom improvement across four trauma-focused treatments, including PE, PCT, CBT, and eclectic therapy [18]. This study also demonstrated no difference in depression symptom improvement between treatment types, with the exception of individuals who dropped out at session three, in which PCT showed statistically significant higher scores than eclectic therapy (p < 0.05). A third study showed that at six weeks, individuals who received PCT therapy had higher odds of PTSD compared to those who received PE therapy (OR = 2.07, 95% CI, 0.99, 4.30, p < 0.05) [18].

**Table 2.** Summary of research studies.

	**Author**	**Location**	**Study Population**	**Methodology**	**Measures**	**Results**	**Impact on Inequities**
**1**	Alvidrez, Shumway, Boccellari, Green, Kelly, & Merrill (2008) [13]	San Francisco, CA San Francisco General Trauma Recovery Center (UCSF TRC)	Study period: 2001–2006 All patients n = 541 Male = 407 (75.1%) Black = 280 (51.7%) Latino = 66 (12.2%) White = 113 (20.8%) Mixed/Other = 83 (15.3%) Mean age (years), SD = 37.0, 11.3 Mean education (years), SD = 12.0, 2.3 Less than HS education = 179 (33.1%) Homeless = 222 (41.0%) Unemployed = 343 (63.4%) Mean monthly income ($) = 1147 TRC services n = 337 Male = 245 (72.5%) Black = 168 (49.9%) Latino = 43 (12.8%) White = 78 (23.1%) Mixed/Other = 48 (14.2%) Mean age (years), SD = 36.4, 11.5 Mean education (years), SD = 12.0, 2.3 Less than HS education = 111 (32.9%) Homeless = 135 (39.9%) Unemployed = 224 (66.5%) Mean monthly income ($) = 1283 Inclusion: injured victim of violent crime presented for emergency medical treatment at SF General Hospital; ≥18 years old; SF resident Exclusion: currently enrolled in mental health or priorly enrolled at TRC; unable to provide consent; no English proficiency; acute psychosis or suicidality; sexual assault victims	Randomized control trial with individuals randomly assigned to receive Trauma Recovery Services vs usual community care	**Outcome Metric** Filed victim compensation claim Received victim compensation **Control/Treatment Group** Analysis of outcomes by TRC vs. usual care	Filed victim compensation claim: TRC = 189 (55.9%) Usual care = 47 (23.0%) p ≤ 0.001 Approved victim compensation claim: TRC = 148 (78.3%) Usual care = 43 (91.5%) p = 0.04	Assignment to TRC services rather than usual care mitigated reductions in application for victim compensation in individuals who were ≤ 35 years, had less than a high school education, or were homeless.
**2**	Alvidrez, Shumway, Kelly, Smart, Gelb, Okin, Merrill, & Boccellari (2008) [14]	San Francisco, CA San Francisco General Trauma Recovery Center (UCSF TRC)	Study period: 2001–2006 n = 329 Male = 236 (71.7%) Black = 163 (49.5%) Latino = 42 (12.8%) White = 72 (23.4%) Mixed/Other = 47 (14.3%) Mean age (years), SD = 36.4, 11.5 Mean education (years), SD = 12.0, 2.3 Homeless = 132 (40.1%) Employed = 33 (10.0%) Median monthly income ($) = 547 Inclusion: injured victim of violent crime presented for emergency medical treatment at SF General Hospital; ≥18 years old; SF resident; client randomly assigned to TRC service Exclusion: currently enrolled in mental health or priorly enrolled at TRC; unable to provide consent; no English proficiency; acute psychosis or suicidality; sexual assault victims	Retrospective cohort study of individuals randomized to TRC services in RCT as described in Alvidrez, Shumway, Boccellari, Green, Kelly, & Merrill (2008) [13]	**Process Metric** Treatment Initiation (≥ 1 session) Case Management Initiation (≥ 1 session) Psychotherapy Initiation (≥ 1 session) **Stratification** Outcomes stratified by demographic, psychiatric diagnosis (PHQ), substance use, mental health treatment history, and acute stress symptoms (Acute Stress Disorder Scale)	238 (72.3%) completed intake 197 (60.0%) treatment initiation 197 (60.0%) received case management 84 (26.0%) received psychotherapy Predictors of treatment initiation Case Management: Higher hyperarousal score Interested in talking to someone Lower avoidance score Psychotherapy: Employed prior to crime Housed No drug use Lower avoidance score	No differences in treatment initiation by gender or race/ethnicity.
**3**	Ghafoori, Hansen, Garibay, & Korosteleva (2017) [15]	Long Beach, CA Long Beach Trauma Recovery Center (LBTRC)	Study period: April 2014 – March 2016 n = 71 Female = 59 (83.1%) Asian / Pacific Islander = 2 (2.8%) Black = 14 (19.7%) Latinx = 31 (43.7%) White = 20 (28.2%) Other = 4 (5.6%) Mean age (years), SD = 35.2, 12.0 No HS diploma = 16 (22.5%) Employed = 18 (25.4%) Income <US$6000/year = 28 (40%) Inclusion: ≥18 years old; English speaking; experienced or witnessed traumatic event; PCL-5 ≥ 33 and PTSD diagnosis Exclusion: acute psychosis; suicidal/homicidal ideation within 1 year of study; hospitalization in prior year for psychiatric issues; substance abuse within 3 months; cognitive impairment; pregnant	Randomized control trial with individuals randomized to receive PCT vs. PE	**Outcome Metric** PCL-5 SDS BSI-18 **Control/Treatment Group** Analysis of outcomes by PCT vs PE treatment	PTSD, depression, anxiety, somatization symptoms showed no statistically significant difference in sessions 3, 6, 9, or 12 in PE vs. PCT p ≥ 0.05 No difference in number of sessions attended in PE vs. PCT p ≥ 0.05 Mixed-effect regression model shows significant effect for PE vs. PCT for PCL-5 score only, F (1, 51.3) = 4.76, p = 0.034	Not assessed
**4**	Ghafoori & Taylor (2017) [16]	Long Beach, CA Long Beach Trauma Recovery Center (LBTRC)	Study period: June 2014–October 2015 n = 27 Female = 27 (100%) Black = 11 (40.7%) Other = 16 (59.3%) 18-24 years = 14 (51.9%) ≥25 years = 13 (48.1%) Graduated HS = 12 (44.4%) Inclusion: vulnerable population; income ≤ federal poverty level; ≥18 years old; English speaking; experienced or witnessed traumatic event; experienced human sex trafficking Exclusion: suicidal/homicidal ideation within 1 year of study; hospitalized in prior year for psychiatric issues; substance abuse within 3 months; cognitive impairment	Retrospective cohort study	**Process Metric** Number of therapy sessions attended **Stratification** Outcomes stratified by TAY (18–24 years) or Older Adult (≥25 years)	66.7% attended ≥ 2 sessions of TAY 61.5% attended ≥ sessions of older adults 64% attended ≥ 2 sessions across all groups No statistically significant in session attendance between TAY and older adults	Not assessed
**5**	Ghafoori, Garfin, Ramírez, & Khoo (2019) [17]	Long Beach, CA Long Beach Trauma Recovery Center (LBTRC)	Study period: April 2017–August 2017 n = 128 Female = 77 (60.2%) Black = 12 (9.4%) Latinx = 79 (61.7%) White = 17 (13.3%) Other = 20 (15.6%) Mean age (years), SD = 11.53, 4.02 Income <US$6000/year = 86 (66.7%) Inclusion: <18 years old, victim of crime/violence; contact with LBTRC staff member for screening, completion of baseline questionnaires Exclusion: active psychosis; brain injury; impaired cognitive functioning	Retrospective cohort study	**Process Metric** Treatment Initiation (≥1 therapy session) Treatment Completion (≥8 therapy sessions) Treatment selection (TF-CBT vs. CCT) **Stratification** Outcomes stratified by demographics, index trauma experienced, emotional and behavior problems (CBCL)	89 (69.5%) treatment initiation 55 (43.0%) treatment completion Predictors of treatment completion: TF-CBT	No differences in treatment initiation or completion by age, gender, race/ethnicity, index trauma, internalizing symptoms, externalizing symptoms.
**6**	Ghafoori, Wolf, Nylund-Gibson, & Felix (2019) [18]	Long Beach, CA Long Beach Trauma Recovery Center (LBTRC)	Study period: April 2014–August 2017 n = 526 Female = (81.09%) Latinx = (55.13%) Mean age (years) = 36.33 Graduated HS = (68.70%) Inclusion: ≥18 years old; victim of crime/violence; contact with LBTRC staff member for screening, completion of baseline questionnaires Exclusion: active psychosis; brain injury; impaired cognitive functioning	Retrospective cohort study	**Process Metric** Drop out (<9 sessions) **Outcome Metric** Change in clinical measures (PCL-5, BSI-18, LEC-5) measured every 3 sessions from baseline to session 12 **Stratification** Outcomes stratified by treatment type: PE, CBT, PCT, or eclectic treatment and drop out (<9 sessions)	PCT lowest proportion of treatment dropout (41.75%) compared to CBT (56.82%) and eclectic (61.05%) p < 0.05 For PTSD, there was no significant difference in pre-post clinical measures across treatment type or dropout For depression, there was no significant difference in pre-post clinical measures across treatment type or dropout, except for those who dropped out at session 3, PCT showed improvement over eclectic therapy p < 0.05	Not assessed
**7**	Ghafoori & Khoo (2020) [19]	Long Beach, CA Long Beach Trauma Recovery Center (LBTRC)	Study period: April 2014–December 2016 n = 163 Female = 137 (84.6%) Black = 33 (20.2%) Latinx = 81 (49.7%) White = 32 (19.6%) Other = 17 (10.4%) Mean age (years), SD = 35.6, 12.5 HS diploma or less = 66 (40.5%) Employed = 50 (30.7%) Income <US$6000/year = 67 (42.1%) Inclusion: ≥18 years old; completion of baseline/pre-test assessment and session 6 assessment; received PE or PCT therapy; met criteria for PTSD; no substance abuse	Retrospective cohort study	**Outcome Metric** Change in clinical measures (PCL-5 and BSI-18) **Stratification** Outcomes stratified by race/ethnicity and treatment type (PCT vs PE)	White participants increased likelihood of probable PTSD at the 6-week compared to the Latinx participants (OR = 0.32, 95% CI, 0.11, 1.00, p < 0.05)* White participants increased likelihood of anxiety at the 6-week compared to the Black participants (OR = 0.07, 95% CI, 0.13, 0.38, p < 0.01), Latinx participants (OR = 0.09, 95% CI, 0.02, 0.42, p < 0.01) and Other participants (OR = 0.05, 95% CI, 0.01, 0.32, p < 0.01)* White participants had increased likelihood of depression at 6 weeks compared to the Other group (OR = 0.17, 95% CI, 0.04, 0.78, p < 0.05)* *adjusted for demographics (employment, education, total no. potential trauma) Individuals in PCT therapy had greater odds of probable PTSD compared to those in PE group (OR = 2.07, 95% CI, 0.99, 4.30, p < 0.05)*	Participants identifying with racial minority groups (black, latinx, and other) had improved clinical measures compared to white participants
**8**	Ghafoori, Hansen, & Garibay (2021) [20]	Long Beach, CA Long Beach Trauma Recovery Center (LBTRC)	Study period: April 2014–March 2016 n = 941 Female = 715 (76.7%) Asian = 24 (3.5%) Black = 141 (20.4%) Hispanic = 364 (52.8%) White = 119 (17.2%) Other = 42 (6.1%) Mean age (years) = 35.87, 12.8 No HS diploma = 179 (31.4%) Income <US$6000/year = 273 (49.6%) Inclusion: ≥18 years; victim of criminal violence; contact with LBTRC staff member for screening; completion of baseline questionnaires Exclusion: active psychosis; brain injury; impaired cognitive functioning	Retrospective cohort study	**Process Metric** Treatment Access (in-person screening interview) Treatment Initiation (≥1 psychotherapy) **Stratification** Outcomes stratified by demographics, as well as predisposing, enabling, and need variables (assessed via LEC-5, PCL-5, BSI-18, WHOQOL-BREF)	524 (55.7%) treatment access 414 (44.0%) treatment initiation Predictors of accessing treatment: Older Less PTSD Predictors of initiating treatment: Higher global severity of distress Poorer quality of life in area of psychological health Better quality of life in area of physical health	No difference in treatment access by gender, race/ethnicity, level of education, household income. No difference in treatment initiation by gender, race/ethnicity, level of education, household income.
**9**	Ghafoori, Matos, & Gonçalves (2022) [21]	Long Beach, CA Long Beach Trauma Recovery Center (LBTRC)	Study period: April 2014–March 2020 n = 1186 Female = 991 (87.9%) Asian = 42 (3.6%) Black = 143 (12.3%) Latinx = 661 (56.9%) White = 172 (14.8%) Other = 143 (12.3%) Mean age (years), SD = 34.39, 11.37 Less than HS = 376 (32.2%) Income <US$12000 = 728 (65.0%) Employed = 365 (31.5%) Inclusion: ≥18 years old; treatment-seeking survivor of interpersonal violence who experienced direct exposure; reporting PTSD or subthreshold PTSD symptoms; contact with LBTRC staff member for screening, completion of baseline questionnaires Exclusion: active psychosis; brain injury; impaired cognitive functioning	Retrospective cohort study	**Process Metric** Pretreatment dropout (no therapy sessions) Postinitiation dropout (1-8 therapy sessions) Treatment completion (≥9 therapy sessions) **Outcome Metric** Change in symptoms (assessed by PCL-5 and BSI-18) **Stratification ** Outcomes stratified by demographics, predisposing characteristics (LEC-5, ATSPPH), enabling factors (WHOQOL-BREF), need factors (PCL-5, BSI-18)	375 (31.6%) pretreatment dropout 332 (28.0%) postinitiation dropout 479 (40.4%) treatment completion Pretreatment dropout predictors: Male White/Black race Unemployed Lower environmental quality of life Post initiation dropout predictors: Younger High school education or less Experience domestic violence Higher social relationships Treatment completion predictors: Female Latinx Employed Experienced sexual abuse Change in symptoms from session 1 vs session 9: Meet criteria for PTSD: 72.6% vs. 32.2%, p < 0.001 Mean PTSD: 44.46 vs. 26.55, p < 0.001 Meet criteria for depression: 68.6% vs. 41.6%, p < 0.001 Mean depressive severity: 66.92 vs. 59.62, p < 0.001 Meet criteria for anxiety: 68.3% vs. 46.1%, p < 0.001 Mean anxiety: 67.43 vs. 59.23, p < 0.001	Individuals who are male, white, black, or unemployed were more likely to dropout prior to treatment initiation. Individuals who are younger and have a high school education or less are more likely to dropout of treatment following initiation. Individuals who are female, Latinx, or employed are more likely to complete treatment.
**10**	Ghafoori, Martinho, Gonçalves, & Matos (2022) [22]	Long Beach, CA Long Beach Trauma Recovery Center (LBTRC)	Study period: April 2014–February 2020 n = 1264 Female = 1037 (87%) Asian = 42 (3.40%) Black = 152 (12.30%) Latinx = 689 (55.7%) White = 196 (15.90%) Other = 157 (12.70%) Mean age (years), SD = 34.67, 11.48 Less than HS = 388 (31.10%) Employed = 384 (31.10%) Inclusion: ≥18 years old; self-identification as survivor seeking help for SA, DVT, or ST; contact with LBTRC staff member for screening, completion of baseline questionnaires Exclusion: missing type of trauma experienced, missing file; active psychosis; brain injury; impaired cognitive functioning	Retrospective cohort study	**Process Metric** Treatment Initiation (≥1 psychotherapy) **Stratification** Outcomes stratified by victims of ST, DV, or SA	58 (56.0%) treatment initiation for ST victims 384 (63.4%) treatment initiation for DV victims 369 (66.5%) treatment initiation for SA victims 811 (64.2%) treatment initiation across all groups No difference in treatment initiation in ST vs. DV vs. SA p = 0.06 Predictors of treatment initiation: Older Female Employed Better social relationships	Being older, female, or employed is associated with treatment initiation
**11**	Simske, Rivera, Ren, Benedick, Simpson, Kalina, Hendrickson, & Vallier (2021) [23]	Cleveland, Ohio Victims of Crime Advocacy and Recovery Program (VOCARP)	Study period: March 2017–December 2018 All patients n = 1432 Male = 838 (58.5%) Black = 714 (49.9%) Hispanic = 119 (8.3%) White = 606 (42.4%) Other = 111 (7.8%) Mean age (years), SD = 36.6, 15.5 Employed = 506 (35.4%) VOCARP service n = 1019 Male = 567 (55.6%) Black = 554 (54.3%) Hispanic = 93 (9.1%) White = 378 (37.1%) Other = 88 (8.6%) Mean age (years), SD = 34.4, 13.7 Employed = 356 (35.0%) Inclusion: presentation to the emergency department for traumatic injury	Prospective/Retrospective cohort study	**Process Metric** VOCARP use Social services used **Outcome Metric** Recidivism (return to ED or clinic for new violence related injury) **Stratification** Traumatic injury from violence or crime withVOCARP service use Traumatic injury from violence or crime without VOCARP service use Traumatic injury not from violence or crime	Predictors of VOCARP service use: Female Single Unemployed Uninsured Services used: Education (criminal justice/victim rights): 974 (95.6%) Financial compensation: 314 (30.8%) Referral to victim service program: 273 (26.8%) Crisis intervention: 228 (22.4%) Emergency shelter: 107 (10.5%) Transportation: 91 (8.9%) No difference in recidivism for VOCARP service users (10.9%) and non-users (8.5%) p = 0.33	Being female, single, unemployed, or insured associated with VOCARP service use
**12**	Simske, Rivera, Ren, Benedick, Simpson, Kalina, Hendrickson, & Vallier (2022) [24]	Cleveland, Ohio Victims of Crime Advocacy and Recovery Program (VOCARP)	Study period: March 2017–December 2018 All patients n = 1432 Male = 838 (58.5%) Black = 714 (49.9%) Hispanic = 119 (8.3%) White = 606 (42.4%) Other = 111 (7.8%) Mean age (years), SD = 36.6, 15.5 Employed = 506 (35.4%) VOCARP service n = 1019 Male = 567 (55.6%) Black = 554 (54.3%) Hispanic = 93 (9.1%) White = 378 (37.1%) Other = 88 (8.6%) Mean age (years), SD = 34.4, 13.7 Employed = 356 (35.0%) Inclusion: presentation to the emergency department for traumatic injury	Prospective/Retrospective cohort study	**Process Metric** VOCARP use **Outcome Metric** Recidivism (return to ED or clinic for new violence related injury) **Stratification** Traumatic injury from violence or crime withVOCARP service use * Use of mental health services vs. not Traumatic injury from violence or crime without VOCARP service use Traumatic injury not from violence or crime	Predictors of VOCARP service use: Female Single Unemployed Uninsured Preexisting mental illness For patients enrolled in VOCARP services use, those who used mental health services had lower rates of recidivism (4.4%) compared to those who did not (11.7%) p = 0.016	Being female, single, unemployed, or insured associated with VOCARP service use

### 3.4. Impact on improving access and outcome inequities for underserved communities

In several of the studies, it was evaluated whether TRC services mitigated disparities in process metrics [14],[17],[20]–[24]. Three studies showed no difference in access, initiation, or treatment completion by age, gender, race, education, or income [14],[17],[20]. Conversely, a later study with the longest study period and largest number of participants (April 2014 to March 2020, n = 1186) showed more complex findings [21]. In this study, individuals who dropped out prior to treatment were more likely to be male, white or black race, and unemployed (p < 0.05), while individuals who dropped out following treatment initiation were more likely to be younger with lower education (p < 0.05). Individuals who completed treatment were more likely to be female, Latinx, and employed (p < 0.05). Another study conducted during the same period with overlapping study participants showed similar results [22]. In this study, individuals who were older, female, or employed were more likely to initiate treatment. Finally, an evaluation in Ohio showed that participants who had experienced a traumatic injury and enrolled in TRC services were more likely to be female, unemployed, and uninsured, compared to those who had a traumatic injury and did not enroll in TRC services [23],[24].

In two studies, the mitigation of disparities in outcomes were assessed [13],[19]. One study demonstrated that rates of victims' compensation application improved with TRC services for those who were younger (p = 0.62), had education (p = 0.78), or did not have housing (p = 0.09) [13]. In comparison, for individuals who received usual services, those who were 35 and younger (p = 0.002), had less than a high school education (p = 0.02), or were unhoused were less likely to file a claim (p < 0.001). A second study assessed changes in PTSD, anxiety, and depression and demonstrated that individuals who identified as Latinx or black had increased likelihood of improvements in PTSD (p < 0.05) and anxiety (p < 0.01) symptoms in comparison to individuals who identified as white [19].

## 4. Discussion

Despite the expansion of the Trauma Recovery Center model to 53 centers in 12 states and millions of dollars allocated by state and federal legislators, there is limited research on the implementation and impact of the TRC model likely due to its recent implementation and limited funding dedicated to evaluation. The 12 studies identified in this scoping review are limited to three Trauma Recovery Centers, with the majority of evidence reported by one center in California. The evidence is predominately observational with few control groups; only two studies used a randomized study design [13],[15]. Results are limited to program evaluations assessing quantitative data rather than mixed methodological or qualitative perspectives from providers and clients. Given that the scoping review is limited to studies at three Trauma Recovery Centers, it is challenging to generalize findings and results must be interpreted within the context of these limitations.

One goal of the TRC model is to improve access to treatment for victims of crime through assertive outreach to individuals who may otherwise be lost to follow-up. Based on the studies identified in this scoping review, the rates of treatment access and initiation for victims of crime referred to TRC services are higher than those referred to non-TRC victim services. Treatment access at the TRC sites included in this study range from 55.7% to 72.3% and treatment initiation rates range from 44.0% to 72.0% [14],[17],[18],[20]–[22]. In comparison, other models of care for victims of crime demonstrate rates of treatment initiation as low as 3.0% in a statewide survey of victims of crime in Pennsylvania [25] and as high as 14.7% in men injured through community violence in California [26].

Second, the TRC strives to provide comprehensive mental health and case management to improve mental health symptoms and social needs. The results from this scoping review suggest that victims of crime who complete at least nine sessions of evidence-based psychotherapy show improvement in psychological symptoms related to PTSD and anxiety [21]. These results are consistent with the literature, demonstrating that one to two crisis intervention sessions do not impact psychological functioning scores in victims of crime [27], but evidence-based cognitive behavioral therapy provided over at least four sessions can improve PTSD symptoms for victims of violent crime [28]. These results are in alignment with the broader literature of interventions for PSTD which demonstrate that evidence-based psychotherapy can decrease short- and long-term symptoms [29]–[31].

Finally, as discussed, the Trauma Recovery Center model was designed to mitigate inequities in victim services. Studies over the last two decades have consistently shown that younger, non-White males experience higher rates of violent crime [32], PTSD and depression following victimization [33], and unmet needs [34], yet are underrepresented in victim services [35]–[38]. In this scoping review, earlier studies based at the original TRC model at UCSF suggest that receiving TRC services reduces gender and race disparities in access to treatment [14]; however, later studies at other sites redemonstrate gender, age, and racial inequities shown in prior non-TRC models of care for victims of crime [21]–[24], which may be reflective of differences in TRC referral base and/or implementation of the model at other sites. Although limited, the studies that assessed the impact of the TRC model on mental health and social needs outcomes show reduction of disparities in applying for victim compensation [13] and anxiety, depression, and PTSD symptoms [19].

Overall, this scoping review of the TRC model finds promising results for treatment engagement and improvement in psychological and social needs, and mixed results regarding inequities in access to services (Table 3). As the TRC model grows, it is critical to invest in future research to expand the evidence to understand its strengths and limitations (Table 4). Research designs that include implementation and context, for instance, comparing hospital-based as compared to community-based TRCs will be helpful additions to the literature. New TRCs may include novel innovations in their implementation of the TRC and/or face barriers to recreating aspects of the model, which may impact patient outcomes. All stakeholder perspectives, including clients, should be incorporated into future research designs. Finally, more research that includes control groups either through randomized controlled trials or quasi-experimental designs will be crucial in filling the knowledge gaps in the evidence base for the TRC model.

## 5. Limitations

Despite using a robust search strategy, articles that did not explicitly state that the intervention was based at a Trauma Recovery Center may have been excluded. Attempts were made to mitigate by including use of grey literature and reviewing publications identified by experts in the field. Furthermore, given that only 12 articles were identified in this scoping review, it is challenging to make any generalizations regarding the impact of TRCs.

**Table 3.** Critical Findings from Scoping Review of the Trauma Recovery Center Model.

**Critical Findings**
Current research limited to three Trauma Recovery Centers – two based in California and one based in Ohio Access to and engagement with care Access rates range from 55.7% to 72.3% Initiation rates range from 44.0% to 72.0% Completion rates range from 40.4% to 43.0% Conflicting results whether disparities in access to and engagement with care is mitigated by the TRC model Mental health outcomes Individuals who complete mental health sessions show improvement in PTSD, anxiety, and depression measures TRC services mitigate impact of non-White race on improvement of psychological symptoms Social needs outcomes Individuals who complete mental health sessions are less likely to experience injury recidivism Individuals who receive TRC services have higher rates of filing for victim compensation TRC services mitigate impact of younger age, less education, and no housing on filing for victim compensation

**Table 4.** Implications for Future Trauma Recovery Center Practice, Policy, & Research.

Future Practice, Policy, & Research
Support and funding for **expansion of research** of Trauma Recovery Centers including: Diversity of sites to better represent variations in funders, available resources, and pre-existing infrastructure Expansion of methodology to include qualitative perspectives all stakeholders, implementation study designs, quasi-experimental designs, and randomized controlled trials Expansion of outcomes to include mitigation of inequities as a primary focus and longer follow-up to allow for evaluation of long-term impact of services

## 6. Conclusions

The results of the scoping review found initial promising evidence for treatment engagement and psychological and social needs outcomes of the TRC model but suggest a need for more extensive research to assess the impact of Trauma Recovery Centers on victims of crime. As a rapidly expanding public health intervention, it is imperative to generate evidence necessary to ensure high quality and equitable access to care for all victims of crime.

## Use of AI tools declaration

The authors declare they have not used Artificial Intelligence (AI) tools in the creation of this article.
